# The Fate Status of Stem Cells in Diabetes and its Role in the Occurrence of Diabetic Complications

**DOI:** 10.3389/fmolb.2021.745035

**Published:** 2021-11-02

**Authors:** Jinyi Xu, Chengguo Zuo

**Affiliations:** State Key Laboratory of Ophthalmology, Zhongshan Ophthalmic Center, Sun Yat-sen University, Guangzhou, China

**Keywords:** diabetic complications, stem cells, mesenchymal stem cells, bone marrow mesenchymal stem cells, stem cells dysfunctions, stem cells quantity changes, stem cells therapy

## Abstract

Diabetes mellitus (DM) is becoming a growing risk factor for public health worldwide. It is a very common disease and is widely known for its susceptibility to multiple complications which do great harm to the life and health of patients, some even lead to death. To date, there are many mechanisms for the complications of diabetes, including the generation of reactive oxygen species (ROS) and the abnormal changes of gas transmitters, which ultimately lead to injuries of cells, tissues and organs. Normally, even if injured, the body can quickly repair and maintain its homeostasis. This is closely associated with the repair and regeneration ability of stem cells. However, many studies have demonstrated that stem cells happen to be damaged under DM, which may be a nonnegligible factor in the occurrence and progression of diabetic complications. Therefore, this review summarizes how diabetes causes the corresponding complications by affecting stem cells from two aspects: stem cells dysfunctions and stem cells quantity alteration. In addition, since mesenchymal stem cells (MSCs), especially bone marrow mesenchymal stem cells (BMMSCs), have the advantages of strong differentiation ability, large quantity and wide application, we mainly focus on the impact of diabetes on them. The review also puts forward the basis of using exogenous stem cells to treat diabetic complications. It is hoped that through this review, researchers can have a clearer understanding of the roles of stem cells in diabetic complications, thus promoting the process of using stem cells to treat diabetic complications.

## Introduction

At present, with the improvement of living standard, the morbidity and incidence of diabetes are increasing rapidly all over the world, projected to affect 10.9% (700 million) people worldwide by 2045 (Saeedi et al., 2019). Diabetes mellitus (DM) is a chronic disease with the volatility of hyperglycemic state caused by the deficiency of insulin secretion or the damage of its biological function, or both (Alberti and Zimmet, 1998; Tripathi and Srivastava, 2006). There are two major forms of diabetes, type 1 diabetes mellitus (T1DM) and type 2 diabetes mellitus (T2DM). T1DM is usually attributed to autoimmune β-cell destruction and characterized by insulin dependence, which occurs mostly in children and adolescents. T2DM is prone to occur in adults and is characterized by insulin resistance (Classification and Diagno, 2020).

Polydipsia, polyuria, polyphagia and emaciation are typical symptoms of DM, which reduce patients’ quality of life. In addition to the harm that “more than three a little” symptoms bring to patients, the complications caused by diabetes are the major cause of death in diabetic patients (Nathan et al., 1993; Intensive blood-glucose c, 1998; Brownlee, 2001). Among all the complications, long-term vascular complications are considered to be the most devastating consequence of diabetes. The vascular complications are due at least in part to chronic hyperglycemia leading to sustained blood vessel damage. They can be divided into microvascular (retinopathy, neuropathy, and nephropathy) and macrovascular (cerebrovascular, coronary artery, and peripheral artery diseases) complications, representing the main contributors to diminished quality of life and increased mortality rate in patients with both T1DM and T2DM (van den Born et al., 2016). Compared with individuals without diabetes, they have a two to four folds increased risk of vascular diseases (Fox et al., 2004). Other chronic complications of diabetes include osteoporosis-associated fracture, diabetic ulcer, sexual dysfunction and et al.

The burden of diabetic complications is directly related to cell, tissue and organ damage, and is also associated with defects in endogenous repair mechanisms. The role of their imbalance in the occurrence and development of the diseases cannot be ignored (Fadini et al., 2017). As we all know, stem cells play an important role in the maintenance of homeostasis and the damaged tissue repair. Therefore, their dysfunctions, or a decline in quantity, may inevitably impair the ability of tissue repair and regeneration, then accelerate the progression of diseases and contribute to diabetic complications. Although diabetes is an extremely complicated disease process with many pathological consequences, some of which have been well established, little is known about whether and how the function and quantity of stem cells, especially MSCs and BMMSCs, are damaged in diabetic microenvironment, and affect the occurrence and progression of diabetic complications.

The aim of this review was to address the specific alteration of the quality and quantity of stem cells, especially MSCs and BMMSCs, in diabetes, briefly discuss the mechanisms of stem cells damage in DM and the relationship between diabetic complications and stem cells changes, and propose a potential modality to manage diabetic complications with stem cell-based therapies. In particular, we explored the properties of diabetic stem cells in terms of 1) dysfunctions, including mobilization, differentiation potential, migration ability, cytokine secretion and et al. 2) the alteration of quantity. By understanding the body of literature devoted to these topics, researchers and practitioners may gain valuable insights into prevention, treatment, and management of diabetic complications. Additionally, it is important to illuminate the changes in the diabetic stem cells microenvironment, which may facilitate the development and improvement of future stem cell-based therapies.

### Mechanisms of Diabetes Induced Injury

Despite the difference in pathophysiology, chronic hyperglycemia is the master switch of diabetic complications in both T1DM and T2DM. As the initiating factor, hyperglycemia ultimately results in endothelial dysfunctions and a variety of cells, tissues and organs damages through different pathways (Forbes and Cooper, 2013; Sena et al., 2013). The underlying molecular mechanisms have been proposed in many researches, including increased polyol pathway flux through aldose reductase, raised advanced glycation end-product (AGE) formation, protein kinase C (PKC) activation, excess glucose flow to hexosamine pathway and et al. (Brownlee, 2001; Forbes and Cooper, 2013). These biochemical pathways can further lead to 1) increase in oxidative stress mainly caused by overproduction of reactive oxygen species (ROS) (Du et al., 2000; Nishikawa et al., 2000; Giacco and Brownlee, 2010) 2) abnormal level of gas transmitters such as nitric oxide (NO), carbon monoxide (CO), and hydrogen sulfide (H2S) (van den Born et al., 2016) 3) increased expression of pro-inflammatory cytokines and pro-coagulant molecules. These factors can then lead to inflammation, endothelial dysfunction and hypercoagulability, which correlate and interact with one another, playing an important role in the development of diabetic complications, especially vascular damages in diabetic patients (Domingueti et al., 2016).

**FIGURE 1:**
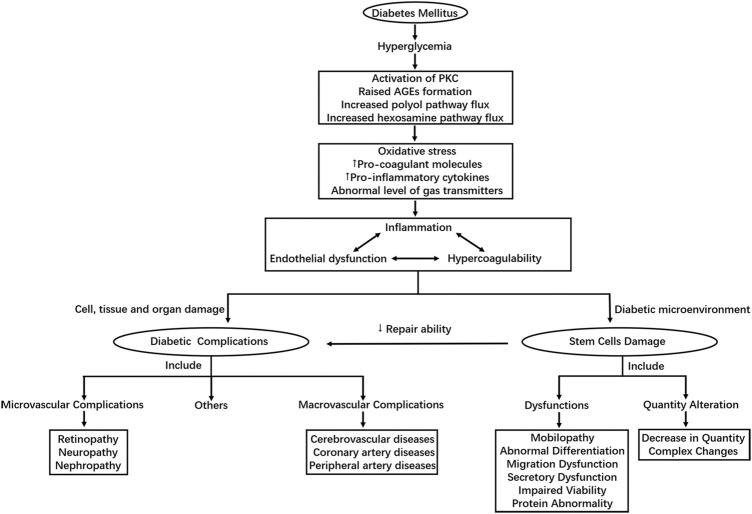
Shows the main mechanism of diabetic complications and stem cell injuries caused by DM.

Under the condition of DM, the homeostasis of body is also damaged through the above mechanisms, leading to changes in cell microenvironment. Long-term exposure to diabetic microenvironment could do great harm to stem cells. The combined effects of oxidative stress, inflammation, abnormal levels of cytokines and et al. initiated by high glucose in diabetic patients may be responsible for the adverse changes in quality and quantity of stem cells (Fadini et al., 2017). In addition to the above mechanisms considering high glucose as the starting factor of the damages, many other mechanisms for the injuries of stem cells induced by diabetes also exist, some of which are mentioned in the review later. Figure 1 shows the main mechanism of diabetic complications and stem cell injuries caused by DM.

### Roles of Stem Cells in Tissue Repair and Homeostasis Maintenance

Stem cells are one of the damaged members which play a significant role in the development of diabetic complications. Stem cells are a population of relatively undifferentiated cells that retain the ability to divide and proliferate throughout postnatal life to provide progenitor cells that can differentiate into specialized cells. Self-renewal and differentiation ability are the most prominent characteristics of stem cells. The ability of self-renewal refers to the process in which stem cells divide symmetrically or asymmetrically to produce at least one daughter cell that retains the characteristics of stem cells (Lodi et al., 2011). The differentiation capacity indicates that stem cells have single or multi-directional differentiation potential and can differentiate into specific or various types of specialized cells. Many studies have reported that these two properties play an important role in tissue regeneration and repair. The ability of self-renewal ensures enough number of stem cells, while the ability of differentiation ensures stem cells to differentiate into damaged cells in time. Under the circumstances of injury and repair, stem cells can proliferate to supplement cells or replace damaged cells (Angelini et al., 2004; Lodi et al., 2011; Chen et al., 2012; Falanga, 2012). Besides directly differentiating into damaged cells in cell replacement manner, stem cells, especially exogenous or nonlocal stem cells, are involved in tissue repair and regeneration mainly through secreting a series of cytokines, as well as a lot of exosomes containing various of miRNAs and lncRNAs. In addition, stem cells have many other characteristics, such as migration and mobilization ability. All of which are of great significance in maintaining tissue homeostasis and repairing damaged tissue. However, when stem cells are damaged under diabetes, whether functionally or qualitatively, their abilities of tissue repair and regeneration are impaired, thus accelerating the progression of diabetic complications.

Among different types of stem cells, mesenchymal stem cells (MSCs), especially bone marrow mesenchymal stem cells (BMMSCs), are most widely applied in treatments for various diseases as a novel potential therapeutic intervention (Charbord, 2010; Berebichez-Fridman and Montero-Olvera, 2018). This can be attributed to their various advantages in biology and medicine. Firstly, they are multipotent and can differentiate into mesodermal lineages, such as adipocytes, osteocytes and chondrocytes. Secondly, MSCs are numerous and can be easily obtained from bone marrow (BM), adipose tissue or umbilical cord tissue. Thirdly, BMMSCs can be derived from autologous, so there is no problem of matching or immune rejection. Fourth, they still have strong immunoregulatory abilities besides their abilities of tissue repair and regeneration. These characteristics make them valuable in a wide range of applications in biological and medical sciences (Naji et al., 2019). Therefore, the review mainly focuses on the impact of diabetes on them. Table 1 summarizes the mechanisms, objects, complications, and the corresponding references of stem cells dysfunctions in diabetes.

**TABLE 1:** Summarizes the mechanisms, objects, complications and the corresponding references of stem cells dysfunctions in diabetes.

Dysfunction	Mechanism	Object	Complication	References
Mobilopathy	disturbed stem cell niches: VCAM1↓SDF-1↓VEGF↓DPP-4↓in BM	HSPCs	various	Orlandi et al. (2010)
	impaired mobilization in response to G-CSF: inability to upregulate DPP-4→SDF-1↓	HSPCs	various	(Fadini et al., 2013a), (Fadini et al., 2013b)
	SNS terminals↑→response of MSCs to β-adrenergic stimulation or G-CSF↓→inability to downregulate CXCL12	HSPCs	neuropathy	Ferraro et al. (2011)
	p66Shc↑Sirtuin1↑→adhesion molecules↑	BM-derived stem cells	neuropathy	Albiero et al. (2014)
	M1/CD169 BMMΦ↑→OSM↑→release of CXCL12 by MSCs↑	Stem cells	various	Albiero et al. (2015)
	APN↓→MC3T3-E1 osteoblastic cells↓→Smad1/5/8 phosphorylation and nuclear localization↓→SDF-1↓	BMMSCs	bone disease	Yu et al. (2015)
Abnormal Differentiation	aberrant angiogenic potential	MSCs	abnormal repair of ischemia	Kim et al. (2015)
	high glucose→adipogenic potential↑	MPCs	vascular disease	Keats and Khan, (2012)
	Nox4↑→ROS↑→adipogenic potential↑	MSCs	vascular disease	Yan et al. (2012)
	differentiation potential toward active fibroblasts↑osteogenic and adipogenic potential↓	Cardiac MSCs	diabetic cardiomyopathy	de Paula et al. (2017)
	T1DM→ERK↓WNT↓p38↑→osteogenic potential↓adipogenic potential↑	MSCs	osteoporosis	Silva et al. (2015)
	T2DM→circulation changes→osteogenic potential↓adipogenic potential↑	MSCs	osteoporosis	Moseley et al. (2018)
	T2DM→BMP-2↓→disturbed BMP signaling pathway→osteogenic potential↓	BMMSCs	osteoporosis	Wang et al. (2013)
	high glucose→osteogenic potential↓	MSCs	osteoporosis	Deng et al. (2018)
	high glucose→osteogenic and chondrogenic potential↓adipogenic potential↑	ASCs	various	Cramer et al. (2010)
	unable to differentiate into mature and functional adipocytesa→hypertrophy of existing mature adipocytes	ASCs	obesity	Barbagallo et al. (2017)
	BMP4↑glucose↑→endothelial and adipogenic potential↑	ASCs	lipodystrophy	Jumabay et al. (2015)
	T1DM→inflammation→Leydig cells↓→testosterone level↓	SSCs	male reproductive disorder	Skurikhin et al. (2017)
	myogenic differentiation ability↓	BMMSCs	various	Jin et al. (2010)
Migration Dysfunction	high glucose	MPCs	neovascularization dysfunction	Keats and Khan, (2012)
	chemokine receptor CXCR4↓	ASCs	various	Kočí et al. (2014)
	high glucose→ERK1/2 and p38 MAPK activities↓→SCF expression↓	CSCs	myocardial infarction	She et al. (2012)
	unknown	ASCs	diabetic ulcer	Cianfarani et al. (2013)
	high glucose→GSK3β↑→chemokine receptor CXCR4↓	ASCs	impaired bone repair	Zhang et al. (2016)
	low-level inflammatory microenvironment→disturbed IL6/STAT3 signaling pathway	MSCs	delayed wound healing	van de Vyver et al. (2016)
Secretory Dysfunction	fibroblast markers↑VEGF-A↓	ASCs	various	Kočí et al. (2014)
	VEGF-A↓IGF-1↓	BMMSCs	various	Jin et al. (2010)
	hepatocyte growth factor↓VEGF-A↓IGF-1↓→keratinocyte and fibroblast proliferation and migration↓	ASCs	diabetic ulcer	Cianfarani et al. (2013)
	IGF-1↓	MSCs	delayed wound healing	Gao et al. (2014)
	T1DM→altered peripheral IGF-1/IGFBP3→dysfunction of colonic stem cells	Stem cells	diabetic enteropathy	D'Addio et al. (2015)
Impaired Viability	high glucose	MSCs	various	(Silva et al., 2015), (Ali et al., 2017)
Protein Abnormality	high glucose→ERK↓WNT↓p38↑→alkaline phosphatase activity↑ collagen synthesis↓	MSCs	various	Silva et al. (2015)
	lactate dehydrogenase release↑	MSCs	various	Ali et al. (2017)
	nestin protein expression↓	Cardiac neural stem cells	diabetic cardiomyopathy	El-Helou et al. (2009)

VCAM1, vascular cell adhesion molecule-1; SDF-1, stromal cell derived factor-1; VEGF, vascular endothelial growth factor; DPP-4, dipeptidylpeptidase-4; BM, bone marrow; HSPCs, hematopoietic stem and progenitor cells; G-CSF, granulocyte colony-stimulating factor; SNS, sympathetic nervous system; MSCs, mesenchymal stem cells; CXCL12, chemokine (C-X-C motif) ligand 12; BMMΦ, bone marow macrophages; OSM, oncostatin M; APN, adiponectin; BMMSCs, bone marrow mesenchymal stem cells; MPCs, mesenchymal progenitor cells; ROS, reactive oxygen species; T1DM, type 1 diabetes mellitus; ERK, extracellular regulated protein kinases; T2DM, type 2 diabetes mellitus; BMP, bone morphogenetic protein; ASCs, adipose derived stem cells; SSCs, spermatogonia stem cells; CXCR4, chemokine (C-X-C motif) receptor 4; MAPK, mitogen-activated protein kinase; SCF, stem cells factor; CSCs, cardiac stem cells; GSK3β, glycogen synthase kinase-3β; IL6, interleukin 6; STAT3, signal transducer and activator of transcription 3; VEGF-A, vascular endothelial growth factor-A; IGF-1, insulin-like growth factor-1; IGFBP3, insulin-like growth factor binding protein 3.

### Stem Cells Dysfunctions in Diabetes

#### Stem Cells Mobilopathy

Mobilization from BM into the circulation is the primary step for stem cells to realize their effects in cell recovery and tissue repair after injury. However, under the condition of DM, stem cells and progenitor cells suffer from “mobilopathy” and are unable to be activated for tissue repair, inducing the progression of diabetic complications.

The BM niches provide the essential microenvironment for maintenance of stem cells function. Hematopoietic stem and progenitor cells (HSPCs) are important kinds of stem cells in BM which can differentiate into mature blood cells with various functions and maintain the homeostasis of blood system. Their abnormalities are related to lowered immunity, difficulty of wound healing and et al. which aggravate the complications. Adhesion molecules, chemokines, cytokines, and proteolytic enzymes form part of the microenvironment and their complex interactions are involved in the regulation of HSPCs mobilization (Lapidot and Petit, 2002). In diabetic mouse, DM compromises the BM niches by down-regulating adhesion molecule VCAM1, chemokine SDF-1, pro-angiogenic cytokine VEGF, peptidase DPP-4 and et al. The disturbed stem cells niche leads to stem cells dysfunctions, thereby, impairing the mobilization of HSPCs (Orlandi et al., 2010). Similarly, in a parallel human group study, HSPCs mobilization in response to subcutaneous injection of human recombinant granulocyte colony–stimulating factor (hrG-CSF) is found to be impaired in patients with DM. This “mobilopathy” is associated with the inability to upregulate protease DPP-4, a regulator of the mobilizing chemokine SDF-1, which is required for the mobilizing effect of hrG-CSF (Fadini et al., 2013a; Fadini et al., 2013b).

In addition, diabetic autonomic neuropathy is involved in the mobilopathy of stem cells. The sympathetic nervous system is prominently involved in BM-derived stem cells trafficking, and the function of sympathetic nervous system in BM is abnormal under the condition of DM. Ferraro et al. found that HSPCs are aberrantly localized in the marrow niche of the diabetic mice, and abnormalities in the number and function of sympathetic nerve termini are associated with this mis-localization. The MSCs innervated by these sympathetic nervous system fibers fail to downregulate chemokine CXCL12 in response to G-CSF or adrenergic stimulation, thus inhibiting the release of HSPCs from BM and making it aberrantly localized in the marrow niche (Ferraro et al., 2011). Furthermore, Diabetic autonomic neuropathy may increase the expression of various adhesion molecules through regulating the expression of 66-kDa protein and Sirtuin 1, leading to BM-derived stem cells mobilization dysfunctions eventually (Albiero et al., 2014).

Stem cells “mobilopathy” in diabetes is also associated with the increase of BM macrophages. Diabetes skews macrophage phenotypes, increases the number of M1/CD169 macrophages in both diabetic mice and DM patients. These macrophages secrete large quantities of soluble mediator oncostatin M, which induce chemokine CXCL12 expression of MSCs *via* a mitogen-activated protein kinase kinase-p38-signal transducer and activator of a transcription 3-dependent pathway (Albiero et al., 2015). Another abnormal pathway is related with adiponectin. Reduction of adiponectin level found in T2DM impairs the mobilization of BMMSCs for bone regeneration, which might be one of the possible mechanisms of bone disease in diabetes. *In vitro* studies found that this is because the reduced adiponectin could not effectively lead to Smad1/5/8 phosphorylation and nuclear localization, thus decreasing the expression of chemokine SDF-1 mRNA (Yu et al., 2015).

#### Abnormal Differentiation

Stem cells have significant plasticity and can differentiate into specialized cells. MSCs are especially multipotent and can differentiate into variety of cell types, including the cells of bone, cartilage, fat and other connective tissue. Within a diabetic microenvironment, however, the differentiation ability of stem cells changes so that they cannot successfully differentiate into the cells required at the injury sites, thus leading to diabetic complications.

Hyperglycemia caused by diabetes can directly act on vascular endothelial cells, leading to vascular dysfunctions, tissue ischemia and many other problems which ultimately result in the complications. Normally, MSCs and mesenchymal progenitor cells can aggregate in the injured area and differentiate into new vascular endothelial cells to restore homeostasis. However, in the case of diabetes, their angiogenic differentiation ability is aberrant and are not able to repair damaged blood vessels (Kim et al., 2015). This is also proved by a group of scientists in 2012. They reported that high glucose drastically increased the differentiation of mesenchymal progenitor cells into adipocytes rather that vascular endothelial cells required for repair (Keats and Khan, 2012). Molecular mechanism is further demonstrated that diabetes can induce the expression of Nox4 which further induce ROS that are essential for the terminal differentiation of MSCs into adipocytes (Yan et al., 2012).

In addition to circulatory vessels, DM can directly affect specific tissues, such as the myocardium, which ultimately progress to diabetic cardiomyopathy (DCM). As one of the most common and severe complication of DM, DCM is characterized by abnormal myocardial metabolism, diastolic cardiac dysfunction, heart failure, cardiomyocyte hypertrophy, cardiac fibrosis and even death in severe patients (Ke et al., 2021). It has been reported that adverse alteration of stem cells found in the heart may be related to the pathogenesis of DCM. Cardiac MSCs are observed with a shift in the differentiation potential toward active fibroblasts that secrete collagen and promote myocardial fibrosis. Reduced capacity for osteogenic and adipogenic differentiation are also reported in DM rats. However, bone or fat is irrelevant to cardiac repair, so whether these changes are associated with DCM remain in doubt (de Paula et al., 2017). Besides differentiation dysfunctions, cardiac stem cells also show abnormalities in migration ability, protein expression, quantity and et al. under diabetes, which are discussed in the later paragraphs. It is worth noting that whether stem cells exist in the heart is controversial at present. Many researchers insist that there are no stem cells in the heart, while some believe a small number do exist (Han et al., 2021). We should be cautious about this issue.

Many patients with diabetes, both T1DM and T2DM, also suffer from an increased risk of osteoporosis-associated fractures according to the influence of DM on skeletal metabolism. In T1DM, patients have reduced bone mineral density (BMD) which may ultimately increase risk of fracture (Kurra et al., 2014). This sustained bone alterations seem to result from a decreased osteoblastic activity. Moreover, osteoporosis is often accompanied by a histologically detectable increase in adipose tissue in the BM. As expected, in T1DM rats, MSCs are observed with the diminished osteogenic and increased adipogenic differentiation. The decreased activity of ERK and WNT, and an increased signaling through p38 may act as its molecular mechanism (Silva et al., 2015). In T2DM, the decreased osteogenic differentiation of MSCs is also significant. Diabetes might alter the circulating factors in microenvironment, which leads to the transformation of MSCs into fat rather than bone, leading to diabetic osteoporosis (Moseley et al., 2018). This changed differentiation ability may also be attributed to the decrease of BMP-2 in BMMSCs, inhibiting the activation of BMP signaling pathway (Wang et al., 2013). However, the influence of T2DM on BMD remains controversial. Studies have reported increased, reduced and even normal BMD of T2DM patients. The conflicting results may partly explained by the combined effects of different quality alteration and degree of decreased osteogenic differentiation ability of MSCs, according to the differences in long-term glycemic control among diabetic patients (Deng et al., 2018).

Most diabetics usually weigh in an abnormal range, either overweight or underweight and some of them also have lipodystrophy. Lipodystrophy is an adipose dysfunction in which your body produces, uses, and stores fat. This kind of disease is mainly related to the obstacle of differentiation of adipose derived stem cells (ASCs) (Cramer et al., 2010; Kočí et al., 2014). Scientists reported that under the condition of diabetes, ASCs lost their ability to differentiate into mature and functional adipocytes. Therefore, DM patients might develop obesity through a hypertrophy of existing mature adipocytes due to failure turnover of adipose tissue (Barbagallo et al., 2017). Based on this, the enhanced expression of BMP4, together with high glucose are further discovered to play a role in increasing endothelial and adipogenic lineage differentiation of ASCs. This promotes adipose vascularity and expression of multiple endothelial cell markers in adipose tissue which changes its normal state (Jumabay et al., 2015).

Moreover, male reproductive disorder are serious complications of T1DM. There are three main cells in the male reproductive system that are related to the disorder, spermatogonia stem cells (SSCs), Sertoli cells and Leydig cells. T1DM results in the inflammation development in the testes and further decreases the number of Leydig cells located in the interstitial tissue of testicles. A great decrease in the number of Leydig cells thus decreases the testosterone level and disturbs the differentiation ability of SSCs. On the contrary, Sertoli cells are hardly affected and form a niche for SSCs, thus minimizing the severity of spermatogenesis disorders in T1DM (Skurikhin et al., 2017).

Above studies *in vivo* indicate that the differentiation abilities of stem cells in diabetic microenvironment are modified. Interestingly, an *in vitro* study found that the properties of stem cells impaired by the diabetic inner environment seems to be irreversible. BMMSCs cultured *in vitro* under normal culture conditions possess decreased myogenic differentiation in induced differentiation experiment, suggesting that the impairment cannot be recovered by *ex-vivo* culture (Jin et al., 2010).

#### Migration Dysfunctions

Cell migration is a crucial process throughout the life of human beings. This is no exception to stem cells. The migration of stem cells is a significant and highly regulated process that enables them to maintain tissue homoeostasis and mediate repair and regeneration. This property, however, can be subverted during the development of diabetes, weakening stem cells abilities of reaching the required position to repair the damaged sites. Untimely repair thus results in the development of diabetic complications (de Lucas et al., 2018).

It has been reported that in patients with diabetes, wounds of tissue or organ are difficult to heal and the prognosis is poor. Patients often suffer from great pain according to pyogenic and ulcerative skin wounds, irreparable vascular injuries and irrecoverable damaged myocardium, et al. All of these are thought to be associated with stem cells migration disorders (Keats and Khan, 2012; She et al., 2012; Cianfarani et al., 2013). Diabetic ulcer, also called diabetic foot is a serious complication of DM, which mainly occurs in the feet of patients and serve as the leading cause of leg amputation. The experimental results show that this was closely related to the decline of migration function of diabetic ASCs (Cianfarani et al., 2013). Similarly, Keats, E. and Z.A. Khan claimed that neovascularization dysfunctions after tissue ischemia was due to reduced cell migration of mesenchymal progenitor cells in hyperglycemia (Keats and Khan, 2012). In addition, for diabetic patients with myocardial infarction, scientists have reported that the inhibition of migration of cardiac stem cells toward the ischemic area post-myocardial infarction was the main cause of the difficulty to recover damaged myocardium (She et al., 2012).

Furthermore, deeper molecular mechanisms of stem cells migration dysfunctions caused by DM have been revealed. The ability of stem cells to express chemokine receptors is related to their ability to migrate to injury sites and mediate damage repair. Under the condition of diabetes, ASCs reduce expression of chemokine receptor CXCR4, causing the dysfunctions (Kočí et al., 2014). Later, Zhang, B, et al. suggested that the suppression of chemokine receptor CXCR4 was due to the activation of its upstream signal transduction pathway, glycogen synthase kinase-3β by high glucose (Zhang et al., 2016). Another research explained the cause of irrecoverable damaged myocardium after myocardial infarction. Diabetic hyperglycemia decreases stem cells factor expression *via* reduction in ERK1/2 and p38 MAPK activities and further inhibits the migration of cardiac stem cells towards the ischemic area post-myocardial infarction (She et al., 2012). Additionally, for MSCs, it has been reported that diabetes creates a low-level inflammatory microenvironment which leads to dysregulation of inflammation-modulated IL6/STAT3 signaling pathway and finally bring about migration dysfunctions. It’s comforting, though, that the dysfunctions can be improved to a certain extent by using recombinant IL-6 (van de Vyver et al., 2016).

#### Secretory Dysfunctions

The paracrine mechanism suggests that stem cells can secrete a variety of substances, such as cytokines and exosomes, promoting different cellular functions including angiogenesis, wound healing, anti-apoptosis and attenuation of fibrosis (Alrefai et al., 2019). However, it has been reported that DM may cause stem cells secretory dysfunctions and result in diabetic complications.

Cytokines released by stem cells play important roles in stimulating endothelial cell migration, inhibiting endothelial apoptosis, promoting angiogenesis and et al. Many researches have reported that under the condition of diabetes, stem cells have a different composition of secretome, containing different levels of angiogenic factors for instance. Either excessive or deficient angiogenesis can lead to diabetic complications (Kim et al., 2015; Ribot et al., 2017). The cytokine vascular endothelial growth factor-A (VEGF-A), an important factor in vascular growth, displays a significant decrease under DM (Jin et al., 2010; Cianfarani et al., 2013). Moreover, the high expression of fibroblast marker, detected by monoclonal anti-human fibroblast surface protein, is associated with this reduced expression of VEGF-A. This suggests the decrease of angiogenesis and the increase of fibrogenesis, which breaks the homeostasis of fiber and vessel formation during wound healing (Kočí et al., 2014).

In addition, lower expression of insulin-like growth factor-1 (IGF-1) has been found under diabetic environment. The delayed wound healing in diabetes is closely related to it (Jin et al., 2010; Gao et al., 2014). According to Cianfarani, growth factors play important roles in skin repair. The decreased release of growth factors results from a reduced paracrine activity of diabetic stem cells at the wound site. This further reduces proliferation and migration of keratinocytes and fibroblasts, resulting in diabetic ulcer (Cianfarani et al., 2013). Interestingly, IGF-1 can also act on stem cells. The level of IGF-1 and its binding protein 3 (IGFBP3) are disrupted in diabetic enteropathy which is common in individuals with long-standing T1DM. And the altered peripheral IGF-1/IGFBP3 results in the dysfunctions of colonic stem cells in diabetic enteropathy (D'Addio et al., 2015).

MSCs also have the ability to secrete exosomes, extracellular nanoparticles containing functional mRNAs, miRNAs, lncRNAs, proteins and lipids (Zhang et al., 2019; van Niel et al., 2018). The therapeutic effect of MSC-derived exosomes has been demonstrated in preclinical studies of DM and the resulting complications. In a rat model of T2DM, intravenous infusion of exosomes from human umbilical cord MSCs can alleviate T2DM by enhancing peripheral insulin sensitivity and inhibiting β-cell apoptosis (Sun et al., 2018). MSC-derived exosomes are also reported to improve functional recovery in mice with diabetic neuropathy and rats with DM-induced myocardial injury *via* suppression of proinflammatory genes and inhibition of TGF-β1/Smad2 signaling pathway, respectively (Fan et al., 2020; Lin et al., 2019). However, so far, most studies have focused on the therapeutic effects of exogenous exosomes. Evidence on the ability of endogenous MSCs to secrete exosomes under DM is limited.

#### Others

In addition to the typical dysfunctions mentioned above, DM also leads to many other stem cells dysfunctions. Many studies have found an impaired viability of MSCs caused by high glucose (Silva et al., 2015; Ali et al., 2017).

Protein abnormality is also one of the factors that are often reported. In diabetic rats, MSCs display increased alkaline phosphatase activity and decreased collagen synthesis through a decreased activity of ERK and WNT, and an increased signaling through p38 resulted from DM. This is associated with abnormal metabolism and regeneration of bone tissue in DM (Silva et al., 2015). It has also been reported that diabetes induce harmful effects on MSCs, increasing the release of lactate dehydrogenase, thus affecting the repair capability (Ali et al., 2017). Besides, El-Helou et al. find that nestin protein expression in cardiac neural stem cells was significantly reduced. The aberrant cardiac neural stem cells phenotype may compromise their biological role and predispose the diabetic heart to maladaptive healing following ischemic injury (El-Helou et al., 2009). Table 2 summarizes the mechanisms, objects, complications and the corresponding references of stem cells quantity alteration in diabetes.

**TABLE 2:** Summarizes the mechanisms, objects, complications and the corresponding references of stem cells quantity alteration in diabetes.

Quantity alteration		Mechanism	Object	Complication	References
Decrease in Quantity	Decreased Proliferation	Bmi1↓	HSPCs	various	Orlandi et al. (2010)
		Unknown	MSCs	various	Kim et al. (2015), Silva et al. (2015), Jin et al. (2010)
		high glucose	ASCs	various	Cramer et al. (2010)
		Unknown	ASCs	diabetic ulcer	Cianfarani et al. (2013)
		high glucose→GSK3β↑→cyclin D1 and CXCR4↓	BMMSCs	various	Zhang et al. (2016)
		high glucose→AGE↑→disturbed RAGE signaling pathway	MSCs	various	Aikawa et al. (2016)
		high glucose→activation of ROS-p38 mediated pathway→chemokines/cytokines↑	MPCs	various	Yang et al. (2010)
		cellular stress↑→p21↑	BMMSCs	various	Gu et al. (2013), Hernandez et al. (2013)
		inflammatory state→TNF-α levels↑	MSCs	delayed fracture healing	Ko et al. (2015)
	Increased Apoptosis	high glucose→ERK↓WNT↓p38↑	MSCs	various	Silva et al. (2015)
		inflammatory state→activation of caspase 3 and caspase 9→TNF-α levels↑	ASCs	various	Cramer et al. (2010)
		high glucose→AGE↑→disturbed RAGE signaling pathway	MSCs	various	Aikawa et al. (2016)
		cellular stress↑→p21↑	BMMSCs	various	Gu et al. (2013), Hernandez et al. (2013)
		inflammatory state→TNF-α levels↑	MSCs	delayed fracture healing	Ko et al. (2015)
		ROS↑	CPCs	cardiomyopathy	Rota et al. (2006)
		microRNA-155↓→FOXO3a↑and nuclear localization→p21↑p27kip1↑	BMPCs	various	Spinetti et al. (2013)
		endoplasmic reticulum stress→p-S6↓→autophagy↑→apoptosis ↑	BMMSCs	various	Meng et al. (2016)
		complement C5a→Fas-associated protein↑BAX/Bcl-2↑	MSCs	various	Zhu et al. (2017)
		metformin→AMPK-mediated mTOR suppression	MSCs	various	He et al. (2018)
	Accelerated Senescence	Bmi1↓	HSPCs	various	Orlandi et al. (2010)
		high glucose	ASCs	various	Cramer et al. (2010)
		high glucose→AGE↑→disturbed RAGE signaling pathway	MSCs	various	Aikawa et al. (2016)
	Increased Cellular Death	unknown	BMMSCs	various	Rezabakhsh et al. (2017)
	Induction of Autophagy	high level of p62	BMMSCs	various	Rezabakhsh et al. (2017)
	Growth Reduction	high glucose	MPCs	vascular disease	Keats and Khan, (2012)
Increase in Quantity	Enhanced Proliferation	unknown	Cardiac MSCs	cardiomyopathy	de Paula et al. (2017)
		BMP4↑glucose↑	ASCs	lipodystrophy	Jumabay et al. (2015)
Complex Changes	Concentration	low blood glucose levels→promote proliferation; high blood glucose levels→inhibit proliferation	MSCs	osteoporosis	Deng et al. (2018)
	Location	BM↓PB↓spleen↑	EPCs	various	Saito et al. (2012)
	Selectivity	T1DM→inflammation→Sertoli cells↓→differentiating c-kit+/CD90+ SSC↑CD51-/CD24+/CD52+ SSC↑sc-kit-/CD90+ SSC↓	SSCs	male reproductive disorder	Skurikhin et al. (2017)
		selectively depleted a subpopulation with a highly vasculogenic transcriptional profile	ASCs	vascular disease	Rennert et al. (2014)

HSPCs, hematopoietic stem and progenitor cells; MSCs, mesenchymal stem cells; ASCs, adipose derived stem cells; GSK3β, glycogen synthase kinase-3β; CXCR4, chemokine (C-X-C motif) receptor 4; BMMSCs, bone marrow mesenchymal stem cells; AGE, advanced glycation end-product; RAGE, receptor for AGE; ROS, reactive oxygen species; MPCs, mesenchymal progenitor cells; TNF-α, tumor necrosis factor-α; ERK, extracellular regulated protein kinases; CPCs, cardiac progenitor cells; BMPCs, bone marrow progenitor cells; BAX, Bcl-2-associated X protein; Bcl-2, B cell lymphoma 2; BMP4, bone morphogenetic protein 4; BM, bone marrow; PB, peripheral blood; EPCs, endothelial progenitor cells; T1DM, type 1 diabetes mellitus; SSCs, spermatogonia stem cells.

### Stem Cells Quantity Alteration in Diabetes

#### Decrease in Quantity

Most of the studies have reported that acute exposure to high levels of glucose under diabetic condition decreases the number of various types of stem cells, leading to insufficiency of stem cells used for repair and regeneration, which strongly relates to different diabetic complications (Keats and Khan, 2012; Skurikhin et al., 2017).

#### Abnormal Proliferation

Inhibition of stem cells proliferation is one of the main reasons for the decrease of stem cells number. Many studies have shown that the proliferation ability of MSCs is obviously impaired within a diabetic microenvironment (Jin et al., 2010; Kim et al., 2015; Silva et al., 2015). It has also been reported that the proliferative potential of ASCs decrease in T1DM (Cianfarani et al., 2013).

This proliferation inhibition may be due to high glucose conditions (Cramer et al., 2010). It is well known that in diabetes, chronic hyperglycemic conditions drive glycation reactions between proteins and glucose or its derivatives, resulting in the formation of AGE. AGE may decrease MSCs proliferation by signaling through receptor for AGE (RAGE) (Aikawa et al., 2016). They may also be responsible for an inhibitory effect on MPCs proliferation by inducing production of chemokines/cytokines *via* activation of ROS-p38 mediated pathway (Yang et al., 2010). Additionally, high glucose may activate glycogen synthase kinase-3beta, which plays an important role in inhibiting the proliferation of BMMSCs by inhibiting cyclin D1 and CXCR4 (Zhang et al., 2016).

Besides high glucose, many other mechanisms have also been proposed. Orlandi et al. show that long-term diabetes decreases the repopulation capacity of HSPCs by reducing the expression of Bmi1 (Orlandi et al., 2010). Another study suggests that the significant decrease of proliferation of BMMSCs is associated with the increase of p21, which is due to increased cellular stress during the pathogenesis of diabetes (Gu et al., 2013; Hernandez et al., 2013). Also, diabetes causes an inflammatory state of the body and increases TNF-α levels, thus reducing stem cells number in new bone area (Ko et al., 2015).

#### Increased Apoptosis

The activation of apoptosis pathway of stem cells by DM is another important reason for the decrease of number (Jin et al., 2010; Baban et al., 2016). Many of the factors mentioned above that reduce the proliferation of stem cells also affect their apoptosis ability. For example, high glucose, p21, TNF-α, AGE and RAGE (Cramer et al., 2010; Stolzing et al., 2010; Gu et al., 2013; Ko et al., 2015). Deeper connections between these factors have also been found. Apoptosis is reported to be induced by high glucose *via* activation of caspase 3 and caspase 9 which is associated with TNF-α (Cramer et al., 2010). High glucose also plays a role in a decreased activity of ERK and WNT, and an increased signaling through p38 (Silva et al., 2015).

Other different mechanisms have also been put forward. The generation of ROS can lead to the apoptosis of cardiac progenitor cells and damage the growth reserve of the heart (Rota et al., 2006). Additionally, the activation of proapoptotic pathway can cause the shortage of CD34(+) bone marrow progenitor cells (BMPCs). Upregulation and nuclear localization of the proapoptotic factor FOXO3a and induction of FOXO3a targets, p21 and p27kip1 play a significant role. Moreover, microRNA-155, which regulates cell survival through inhibition of FOXO3a, is downregulated in diabetic CD34(+)-BMPCs and inversely correlated with FOXO3a levels (Spinetti et al., 2013).

In 2016, it has been reported for the first time that endoplasmic reticulum stress induces apoptosis of BMMSCs, which is mediated by autophagy associated with the decrease of p-S6 (Meng et al., 2016). Later, Zhu et al. found that DM induces MSCs apoptosis through complement C5a-dependent up regulation of Fas-associated protein with death domain and the Bcl-2-associated X protein (BAX)/B cell lymphoma 2 (Bcl-2) ratios (Zhu et al., 2017). More recently, it is suggested that when metformin is used to enhance glucose control in diabetic patients, it might induce MSCs apoptosis through AMPK-mediated mTOR suppression, while high glucose (standard glucose control) can significantly reverse its adverse reactions in an AMPK-mTOR pathway dependent manner (He et al., 2018; He et al., 2019).

#### Others

In addition to inhibiting proliferation and promoting apoptosis, a few reports have suggested that diabetes can reduce the number of stem cells by some other mechanisms.

Long-term diabetes may accelerate the senescence of HSPCs by reducing the expression of Bmi1 (Orlandi et al., 2010). The accelerated aging of stem cells also relates to the increase of glucose in T2DM and the change of AGE and RAGE (Cramer et al., 2010; Stolzing et al., 2010).

It has been reported that diabetic serum is found to induce a higher cellular death rate and decrease human BMMSCs angiogenic properties by the induction of autophagy signaling which is marked by high level of p62 (Rezabakhsh et al., 2017). Additionally, Keats and Khan found that MPCs show a transient reduction in growth upon glucose challenge (Keats and Khan, 2012).

### Increase in Quantity

Although most of the literatures report the damage of diabetes to the number of stem cells, still, some have reported that diabetes increases their quantity.

A higher number of cardiac MSCs in DM rats has been observed *in vitro*, which is associated with a significantly higher proliferative rate. However, the confirmation of this assertion requires more experiments to evaluate their mitotic rate *in vivo* (de Paula et al., 2017). Additionally, cautiousness is required on the existence of stem cells in the heart. For ASCs, this enhanced proliferative rate results from the change of glucose and BMP4 (Jumabay et al., 2015).

### Complex Changes in Quantity

Moreover, some studies have reported complex changes in the number of stem cells under DM condition.

The number of stem cells is related to the level of glucose. A certain degree of hyperglycemia promotes MSCs proliferation, whereas high blood glucose levels (>10%), which reflects poor glycemic control, significantly inhibits MSCs proliferation (Deng et al., 2018).

Additionally, the location of stem cells may also be connected with their quantity. The number of circulating endothelial progenitor cells (EPCs) significantly decreases in BM and peripheral blood, but paradoxically increases in spleen under diabetic conditions. This may be attributed to the damage of EPCs production in BM and the reduction of mobilization of spleen EPCs (Saito et al., 2012). However, Keats and Khan reported that EPCs were resistant to the effects of high levels of glucose, even following chronic exposure (Keats and Khan, 2012).

A study further claimed that this quantity change was not overall or random. For ASCs, diabetes selectively depletes a subpopulation with a highly vasculogenic transcriptional profile, thus impairing new blood vessel formation and wound healing (Rennert et al., 2014). Among the male reproductive disorders caused by T1DM, the proliferation of SSCs plays a critical role. The pronounced inflammatory component of T1DM slightly decreases the number of Sertoli cells which can regulate the SSCs *via* secretion of various factors. Thus, the number of SCCs with c-kit-/CD90+ immunophenotype significantly decreases and the number of differentiating c-kit+/CD90+ SSC and CD51-/CD24+/CD52+ SSCs significantly increases (Skurikhin et al., 2017).

### Stem Cell-Based Therapy for Diabetic Complications

We can see that diabetes indeed does damage to the endogenous stem cells of the body in all aspects, which leads to severe diabetic complications. Therefore, it is possible that exogenous stem cells can be used to treat the complications. Some studies have reported the effectiveness of the treatment basic *in vitro* experiments and animal experiments. But to the best of our knowledge, existing clinical studies are of low quality and none has proven clinical safety nor efficacy (Li et al., 2021). Here, we briefly put forward some therapies, hoping to inspire more researchers to improve and perfect the therapeutic methods of diabetic complications.

Diabetes damages stem cells from quality and quantity. In most cases, the number of stem cells decreases under DM. Therefore, supplementing exogenous stem cells can relieve the damage of diabetes on the quality of stem cells. Additionally, stem cell dysfunctions caused by diabetes can also be relieved through the normal function of exogenous stem cells. Three specific mechanisms might play a role, including migration, differentiation and secretion ability. Firstly, stem cells undergo homing and further migrate to the injured site, which may be due to chemoattraction mediated by cell surface receptors (Teo et al., 2012). Secondly, exogenous stem cells can differentiate into multiple cell types to replace damaged tissue and induce functional recovery (Aggarwal and Pittenger, 2005). Thirdly, stem cells can secrete growth/bioactive factors and exosomes, which may have potential positive effects on local and systemic physiological processes (Raffaghello et al., 2008). It is important to note that various mechanisms exist in the therapeutic potential of stem cells, above mentioned might not be generally true for all stem cell therapies.

### Microvascular Complications

The microvascular complications of diabetes mainly include retinopathy, neuropathy, and nephropathy. These three major microvascular complications seriously affect the quality of life of diabetic patients and even threaten their lives. Stem cells seem to be an effective long-term treatment option for these complications due to their repair and regeneration potential.

It has been reported that stem cells may delay the progression and alleviate the symptoms of diabetic retinopathy through secretion and differentiation abilities (Scalinci et al., 2011; Davey et al., 2014). Recent studies on MSCs, EPCs and ASCs have shown that cell-based therapy may be a feasible option to prevent neurovascular injury and promote retinal regeneration (Megaw and Dhillon, 2014; Gaddam et al., 2019). However, current authoritative studies mainly focus on basic researches and animal studies. Although these researches have tentatively confirmed the effectiveness of cell regeneration therapy and consolidated the foundation of preclinical research in this field, the stem cell therapy has not yet been widely accepted in clinical practice (Ludwig et al., 2019; Li et al., 2021).

Effectiveness for reversing various manifestations of experimental diabetic neuropathy has also been proved through attempts for treating experimental diabetic neuropathy with BM-derived stem or progenitor cells. In previous studies, MSCs transplantation has been reported to have therapeutic effects on diabetic neuropathy through paracrine actions of growth factors (Shibata et al., 2008). Additionally, MSCs transplantation are able to improve diabetic neuropathy through direct peripheral nerve angiogenesis, neurotrophic effects, and restoration of myelination (Han et al., 2016). More recently, application of MSCs-derived exosomes to diabetic mice cast light on the feasibility and efficacy of the exosome-based therapy for diabetic neuropathy (Fan et al., 2021).

For the treatment of diabetic nephropathy, some studies have reported that in the presence of certain growth factors, embryonic stem cells can differentiate into renal cells and play a therapeutic effect on diabetic neuropathy (Kim and Dressler, 2005; Narayanan et al., 2013). iPSCs have also been successfully differentiated into renal cells (Song et al., 2012). Moreover, the role of MSCs in the treatment of diabetic neuropathy is prospective. MSCs have been introduced into diabetic rats to repair renal damage and regenerate insulin-secreting cells (Ezquer et al., 2008). MSCs can also protect the kidney by stimulating the regeneration microenvironment through paracrine action (Ezquer et al., 2015). It has been further demonstrated that BMMSCs treatment are able to regulate the serum level of insulin, hemeoxygenase-1, AGE, and glucose with recovery in renal function (Hamza et al., 2017).

### Macrovascular Complications

Diabetic patients are prone to atherosclerosis, and eventually develop into macrovascular complications. Vascular stem cells have the ability to differentiate into EPCs, which is a potential target for the treatment of diabetic macrovascular complications (Peng et al., 2018).

## Discussion

Diabetes causes various stem cells dysfunctions such as mobilopathy, abnormal differentiation, migration dysfunctions, secretory dysfunctions and et al. through different molecular pathways. DM also results in abnormal changes in the number of stem cells, which may due to abnormal proliferating rate, increased apoptosis and et al. These may further lead to diabetic complications, which are the main cause of high mortality in patients with diabetes.

Fortunately, in the past few years, the role of stem cells in the treatment of diabetic pathology and related complications has been widely studied. The ability of stem cells to regenerate and differentiate makes it possible for researchers to explore their potential in treating diabetic complications. It has been suggested that normal functional exogenous stem cells might improve microvascular complications and macrovascular complications, which are the major complications of DM. This therapeutic approach not only helps to overcome the limitations of modern treatment, but also provides a new way for long-term treatment of diabetic complications. Therefore, the use of exogenous stem cells in the treatment of diabetic complications is very promising, worthy of further exploration. However, it can’t be denied that stem cells therapy for diabetic complications is still in the imperfect stage of exploration. Extensive research is needed to establish a standard procedure for stem cells therapy of diabetic complications. We hope that this review can provide an in-depth understanding of the association between stem cells and diabetic complications, pave the way for novel insights into the mechanisms underlying diabetes complications, and inspire more researchers to explore new methods for the treatment of diabetic complications.
